# High-Fat-Diet-Induced Kidney Injury in Rats: The Role of Tart Cherry Supplementation

**DOI:** 10.3390/antiox14091102

**Published:** 2025-09-10

**Authors:** Ilenia Martinelli, Proshanta Roy, Vincenzo Bellitto, Maria Vittoria Micioni Di Bonaventura, Carlo Cifani, Seyed Khosrow Tayebati, Daniele Tomassoni

**Affiliations:** 1 School of Pharmacy, University of Camerino, 62032 Camerino, Italy; ilenia.martinelli@unicam.it (I.M.); mariavittoria.micioni@unicam.it (M.V.M.D.B.); carlo.cifani@unicam.it (C.C.); 2 School of Biosciences and Veterinary Medicine, University of Camerino, 62032 Camerino, Italy; proshanta.roy@unicam.it (P.R.); vincenzo.bellitto@unicam.it (V.B.)

**Keywords:** high-fat diet, obesity, kidney, antioxidant, tart cherry, inflammation, transient receptor potential channel

## Abstract

The kidney plays a crucial role in the complex inter-organ communication that occurs during obesity, leading to the development of oxidative stress, inflammation, and fibrosis. Dysfunction of the transient receptor potential (TRP) ion channels contributes to this pathophysiology. This study was designed to evaluate the effects of antioxidant-rich fruit tart cherry (*Prunus cerasus* L.) on kidney morphology and protein expression in rats with diet-induced obesity (DIO). Methods include histological staining and immunohistochemical and Western blot assays. Obese rodents were fed with seed powder (DS) and seed powder plus juice (DJS) of the tart cherry. Results demonstrated that rats fed a high-fat-diet (HFD) showed a significant reduction in renal expression of the pro-inflammatory cytokines interleukin-1 beta (IL-1β) and interleukin-6 (IL-6) following tart cherry supplementation. Furthermore, the study provided evidence that TRP channels, specifically TRP canonical 1 (TRPC1) and TRP melastatin 2 (TRPM2), were significantly upregulated in obese animals (*p* < 0.05 vs. CHOW rats) and markedly downregulated following tart cherry supplementation (*p* < 0.05 vs. DIO rats). In conclusion, these TRP proteins offer new insights for identifying targets and biomarkers for developing therapeutic strategies against HFD-induced renal damage, characterized by glomerulosclerosis, fibrosis, and inflammation. Tart cherries supplementation exerted a protective effect on the kidneys by reducing protein oxidation and pro-inflammatory cytokine expression.

## 1. Introduction

Obesity may predispose individuals to chronic kidney disease (CKD) directly through the production of adiponectin, leptin, and resistin, as well as its association with the histopathological features of obesity-related glomerulopathy. It also contributes indirectly to kidney damage through its well-documented complications, such as atherosclerosis, hypertension, and type 2 diabetes. The direct effects of obesity on the kidneys include glomerular hyperfiltration, increased renal plasma flow, microvascular stretching, activation of the renin–angiotensin–aldosterone system, altered secretion from adipose tissue, abnormal lipid metabolism, and heightened insulin production and resistance. These factors collectively promote endothelial dysfunction, inflammation, oxidative stress, fibrosis, and proteinuria in the kidneys [1,2,3,4]. Additionally, the first renal complication linked to obesity was identified as renal cell carcinoma [5], with renal cell destruction observed in the presence of abnormal lipid metabolism and chronic inflammation [6]. Among the various inflammatory mediators, pro-inflammatory cytokines, such as interleukin-1β (IL-1β) and interleukin-6 (IL-6), are involved in the development and progression of nephropathy. Moreover, diabetes triggers the activation of immune cells and increases the expression of pro-inflammatory cytokines within the kidney, leading to the release of more inflammatory mediators and chemokines that recruit further immune cells. Furthermore, activated resident renal cells also produce additional pro-inflammatory signals, thus sustaining inflammation and kidney damage [7]. Children with severe obesity are at a higher risk of developing early renal abnormalities, including decreased kidney function, and early markers of renal damage [8]. Therefore, the prevention and management of obesity are critical strategies for both preventing the onset and slowing the progression of CKD.

Given the established link between transient receptor potential (TRP) channels and metabolic diseases, it is important to examine their role in mediating intracellular and extracellular stimuli, as well as their contribution to ion homeostasis.

TRP channels are a group of cation channels that influence intracellular Ca^2+^ dynamics [9] and are ubiquitously expressed in mammalian tissues, including adipose tissue. Studies have associated TRP channels with obesity [10]. Although TRP channels might be regarded as therapeutic targets for obesity due to the inhibitory effects of their agonists on body weight and adiposity, the precise role of TRP channels in the progression of obesity by controlling the function of adipose tissue has not been explored. Among the TRP family, certain members, such as transient receptor potential canonical 1 (TRPC1) [11,12], transient receptor potential canonical 6 (TRPC6) [13], and transient receptor potential melastatin 2 (TRPM2) [14], have been implicated in obesity, oxidative stress, and kidney diseases [15,16]. Preceding discoveries and current literature on TRPC1, TRPC6, and TRPM2 highlighted in the kidney their pathophysiological importance. Since they appear to be tangled in obesity, diabetes and hypertension, they could be new therapeutic targets, and the manipulation of these molecules could be a approach to prevent or treat the damaging effects of consequent diseases, such as acute kidney injury and CKD [17]. In addition to the modulation of TRP channels, the consumption of anthocyanin-rich foods with antioxidant properties may also contribute to a lower incidence of kidney diseases.

Anthocyanins, phenolic pigments belonging to the flavonoid family, found in red-, blue-, and purple-pigmented fruits and vegetables, are recognized for their antioxidant and anti-inflammatory properties. Nutritional and health claims have substantially increased the demand for tart cherries in the food industry and have generated specific interest in research in the preservation, stability, and bioavailability of phytochemical content in tart cherry (*Prunus cerasus* L.) after processing. A growing body of evidence supports the anti-inflammatory and antioxidant effects of tart cherries [18]. Tart cherries contains high levels of anthocyanins, flavonoids present in red, blue, and purple fruits and vegetables. The compounds of these plants changed in vitro the metabolism of lipid, and in vivo reduced the dyslipidemia [19].

Obesity is often associated with dyslipidemia, hypertension, impaired glucose tolerance, and insulin resistance, all of which contribute to an increased risk of renal diseases. Rats under diet-induced obesity (DIO) provided a useful animal model sharing many pathophysiological features with human obesity [20]. In this context, a high-fat-diet (HFD) represents a major contributor to the development of obesity in modern societies. Therefore, a DIO model is instrumental in improving our knowledge of how dietary factors contribute to kidney alterations and the metabolic disturbances related to obesity. The aim of the study was to evaluate in obese rats the potential relationships among HFD, kidney damage, and inflammatory processes involving TRP channel activity. Moreover, the study evaluated the possible protective effects of tart cherry seeds and juice supplementation in DIO rats.

## 2. Materials and Methods

### 2.1. Animal and Blood Parameters

Kidney samples were collected from the same male Wistar rats used in the study by [21]. Institutional Guidelines, conforming with the Italian Ministry of Health (protocol number 1610/2013) and associated guidelines from the European Communities Council Directive, were followed. Animals were divided into two groups: CHOW rats (fed a standard diet, 7% fat) and DIO rats (HFD, 45% fat). The effects of *Prunus cerasus* L. supplementation were assessed in DIO animals. These rats received either tart cherry seeds alone (DS) or a combination of tart cherry seeds and juice (DJS). The concentration of anthocyanins tested, the preparation of seed powder and juice from tart cherries, and their composition have been already described elsewhere [21,22] and detailed in the Supplementary Materials. After 17 weeks of being fed a HFD, animals were sacrificed. Body weight, blood parameters, and systolic blood pressure were previously reported [21,22,23,24,25,26,27,28]. While body weight gain was not significantly different between DS, DJS, and DIO groups, both supplemented groups (DS and DJS) showed a reduction in systolic blood pressure compared to untreated DIO rats. The consumption of tart cherries counteracted hyperglycemia but had no effect on hyperinsulinemia. Moreover, triglyceride levels were significantly reduced in tart-cherry-treated groups compared to the DIO control rats [21,22,23,24,27,28]. Additional methods are available in the Supplementary Materials (Supplementary File S1).

Additional methodological details are provided in the Supplementary Materials (Supplementary File S1).

### 2.2. Morphological Aspects

The kidneys were embedded into paraffin and cut into 8 μm sections for Masson’s trichrome and periodic acid–Schiff (PAS) staining. Glomerular injury score (GIS) was measured in Masson’s-stained sections as previously detailed [29], and data were expressed as fold relative to control.

### 2.3. Immunohistochemistry and Western Blot Analysis

The immunohistochemistry (IHC) and Western Blot (WB) procedures and analysis were conducted as previously described [25,27]. The primary antibodies used for IHC were also incubated for WB: anti-IL-1β and anti-IL-6 (both from Santa Cruz Biotechnology, Inc., Dallas, TX, USA), anti-TRPC1, anti-TRPC6 and anti-TRPM2 (all from Alomone Labs, Ltd., Jerusalem, Israel), anti-caspase3 (Cell Signaling Technology, Danvers, MA, USA). β-actin (Sigma-Aldrich Co., St. Louis, MO, USA) was used as a loading control. Moreover, the protein carbonyl levels were analyzed using the OxyBlot protein oxidation detection kit (Merck-Millipore, Burlington, MA, USA), as detailed elsewhere [25,27,29]. The mean intensities of immunostaining and densitometric analysis of the bands were recorded.

### 2.4. Data Analysis

The statistical significance of the differences was performed with the GraphPad Prism (version 8) program using analysis of variance (ANOVA) followed by Tukey’s post hoc test. Data were presented as mean ± standard error of mean (SEM). Statistical significance was set at *p* < 0.05.

## 3. Results

### 3.1. Kidney Weight and Morphology

**Figure 1:**
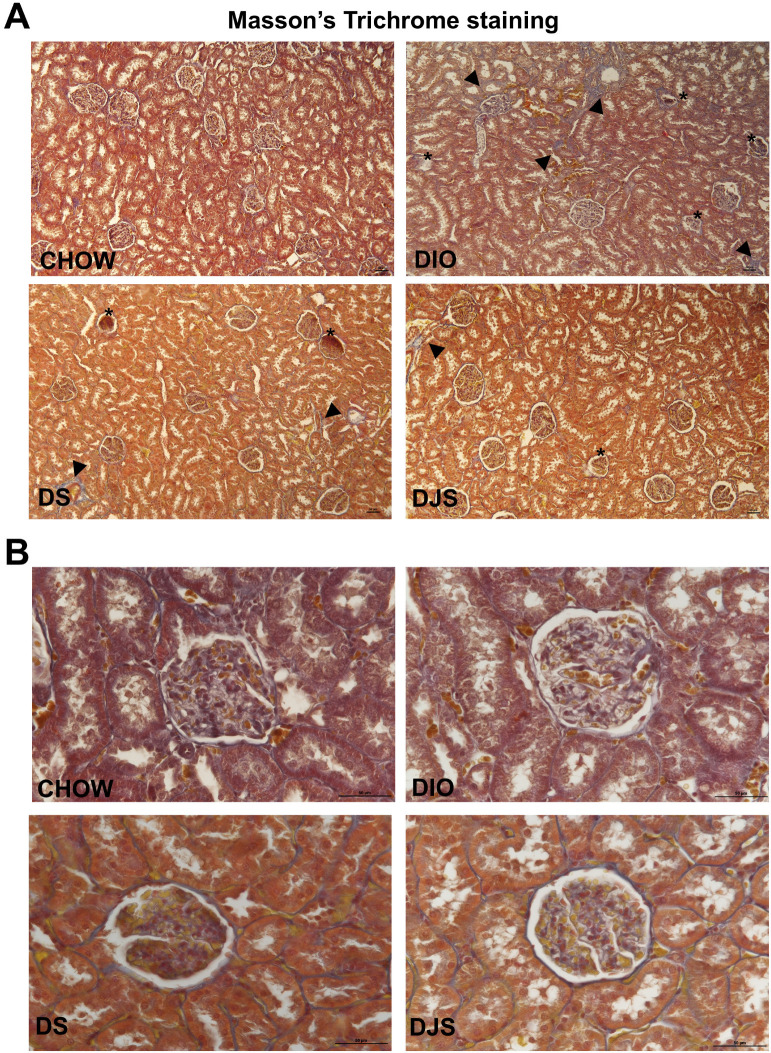
Renal Morphology. Representative pictures of Masson’s trichrome staining. (**A**): Magnification 10×, calibration bar: 50 μm. (**B**): Magnification 40×, Calibration bar: 50 μm. CHOW, rats fed with standard diet; DIO, rats fed with high-fat diet; DS, DIO rats supplemented with tart cherry seeds; DJS, DS rats supplemented with tart cherry juice. Arrow heads: connective tissues deposition around the tubular portions; asterisks: damaged corpuscles.

**Figure 2:**
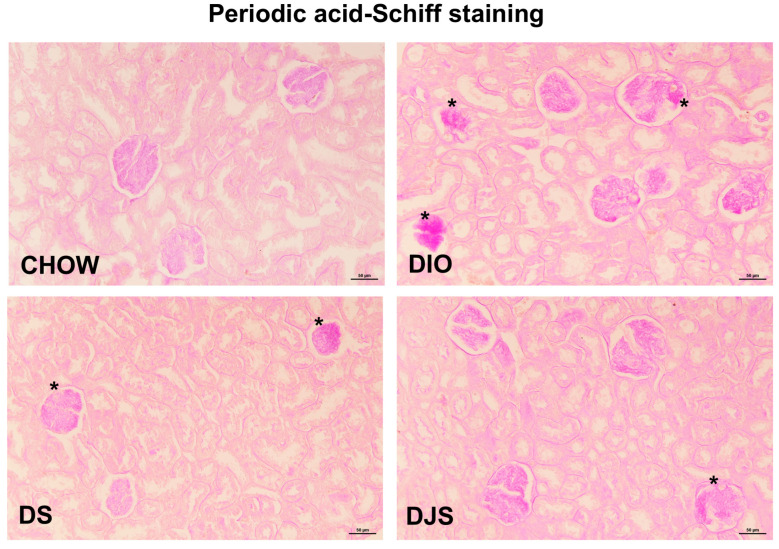
Renal morphology. Representative pictures of periodic acid–Schiff (PAS) staining. Magnification 20×, calibration bar: 50 μm. CHOW, rats fed with standard diet; DIO, rats fed with high-fat diet; DS, DIO rats supplemented with tart cherry seeds; DJS, DS rats supplemented with tart cherry juice. Asterisks: damaged corpuscles.

No significant difference was found in kidney weight across any of the experimental groups (Table 1). Masson’s trichrome staining of kidney sections showed morphological alterations in DIO compared to CHOW, indicating damage at the level of the glomeruli as well as both proximal and distal convoluted tubules (Figure 1A). The renal corpuscles showed signs of damage, including collapsed glomeruli and increased deposition of connective fibers surrounding the tubules. No significant difference was shown in the capsular and glomerular tuft volumes (Table 1 and Figure 2). The glomeruli exhibited global sclerosis, characterized by a loss of cellularity, expansion of the mesangial matrix, and an increased deposition of connective tissue around the parietal cell membrane (Figure 1B). The kidneys of DIO rats were characterized by interstitial fibrosis, tubular atrophy, and glomerular shrinkage. Interstitial fibrosis was evidenced by increased connective fiber deposition around the damaged tubules (Figure 1A).

The interstitial fibrosis and tubular atrophy of DIO rats were less pronounced in DIO rats supplemented with tart cherry seeds and juice (Figure 1A). The GIS was elevated in DIO rats compared to CHOW controls. However, a significant decrease in GIS was recorded in DJS, but not in DS rats, compared to the control DIO rats (Table 1). Similar alterations were evident in sections of the kidney processed for PAS staining, where DIO rats showed a clear glomerular atrophy, highlighted by more dye deposition, compared to the CHOW group (Figure 2).

### 3.2. Oxidative Stress and Apoptosis

**Figure 3:**
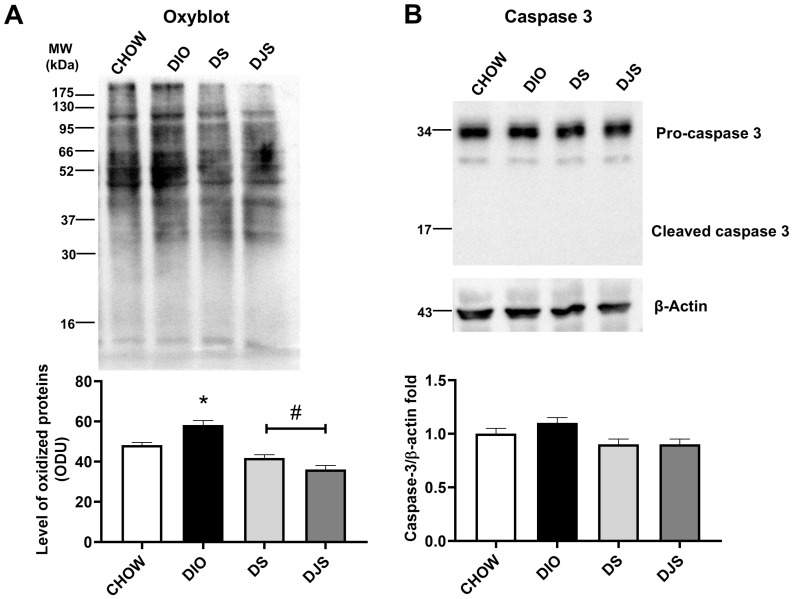
Oxidative stress and apoptosis. (**A**) Oxyblot in kidney samples and the graph shows the level of oxidized proteins expressed as arbitrary optical density unit (ODU). (**B**) Kidney lysates were immunoblotted with anti-caspase 3. The graph shows the densitometric ratios of bands and β-actin expression, used to normalize the data. CHOW, rats fed with standard diet; DIO, rats fed with high-fat diet; DS, DIO rats supplemented with tart cherry seeds; DJS, DS rats supplemented with tart cherry juice. Data are mean ± SEM. *: *p* < 0.05 vs. CHOW rats; #: *p* < 0.05 vs. DIO rats.

The Oxyblot kit revealed an increase in oxidate proteins in the DIO rats compared to CHOW, and a decrease in DS and DJS groups (Figure 3A). The glomerulus sclerosis in DIO rats compared to CHOW was not related to an increase in the apoptotic process. Indeed, WB for caspase 3 showed the same level of pro-caspase 3 among the different groups, without activation of cleaved caspase 3 (Figure 3B).

### 3.3. Pro-Inflammatory Cytokines Expression

**Figure 4:**
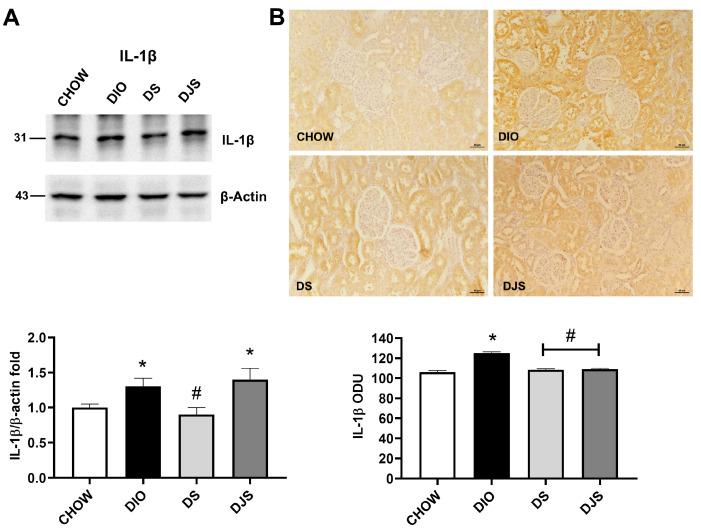
Inflammation. (**A**) Kidney lysates were immunoblotted with anti-interleukin-1β (IL-1β). The graph shows the densitometric ratios of bands and β-actin expression, used to normalize the data. (**B**) Immunohistochemical representative pictures of kidney sections processed for IL-1β. Graphs indicate the mean intensities of the area immunoreaction of IL-1β. Scale bar 50 µm. CHOW, rats fed with standard diet; DIO, rats fed with high-fat diet; DS, DIO rats supplemented with tart cherry seeds; DJS, DS rats supplemented with tart cherry juice. Data are mean ± SEM. *: *p* < 0.05 vs. CHOW rats; #: *p* < 0.05 vs. DIO rats.

**Figure 5:**
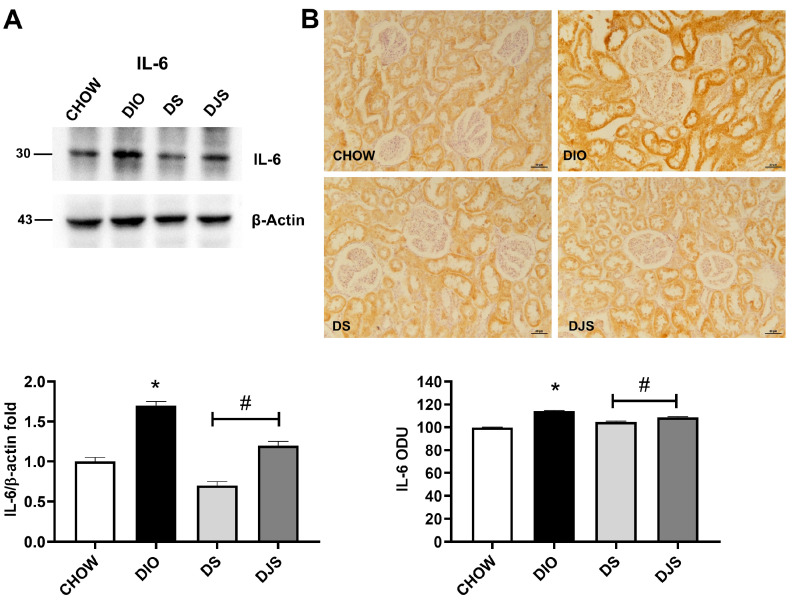
Inflammation. (**A**) Kidney lysates were immunoblotted with anti-interleukin-6 (IL-6). The graph shows the densitometric ratios of bands and β-actin expression, used to normalize the data. (**B**) Immunohistochemical representative pictures of kidney sections processed for IL-6. Graphs indicate the mean intensities of the area immunoreaction of IL-6. Scale bar 50 µm. CHOW, rats fed with standard diet; DIO, rats fed with high-fat diet; DS, DIO rats supplemented with tart cherry seeds; DJS, DS rats supplemented with tart cherry juice. Data are mean ± SEM. *: *p* < 0.05 vs. CHOW rats; #: *p* < 0.05 vs. DIO rats.

The inflammatory response characterized by the augmentation of cytokine was found in DIO rats when compared with CHOW rats. WB analysis of IL-1β and IL-6 revealed bands of approximately 31 kDa and 30 kDa, respectively. Both cytokines were upregulated in the DIO rats compared to CHOW, with a decrease in the DS compared to DIO rats (Figure 4A and Figure 5A). A significant decrease in IL-6 expression was also found in the DJS group (Figure 5A). The immunohistochemical analysis of inflammatory markers performed for IL-1β and IL-6 showed specific immunoreaction in the cortical region of the kidney (Figure 4B and Figure 5B). In particular, the IL-1β immunoreaction was evident in tubular cells and within the glomeruli (Figure 4B), while the IL-6 was expressed only at the level of the tubular cells (Figure 5B).

Immunohistochemical analysis for IL-1β revealed the diffuse expression of the protein in the kidneys of CHOW and DIO rats at the level of the proximal and distal convoluted tubules (Figure 4B). In DIO rats, IL-1β expression increased at the level of the glomeruli, probably in the mesangial cells, and at the basal level of the proximal and convoluted tubules (Figure 4B). This evidence indicated inflammatory processes, particularly in the tubular portion of the nephron of DIO rats (Figure 4B). Immunohistochemical analysis of IL-6 revealed low expression of the protein in the kidneys of CHOW rats (Figure 5B), while the expression increased in the distal convoluted tubules of DIO rats (Figure 5B). These findings indicated inflammatory processes, particularly affecting the tubular portion of the nephron of DIO rats, which may contribute to impaired reabsorption. The expressions of IL-1β and IL-6 decreased in both the DS and DJS rats as measured by densitometric analysis (Figure 4B and Figure 5B).

### 3.4. TRP Channels Expression

**Figure 6:**
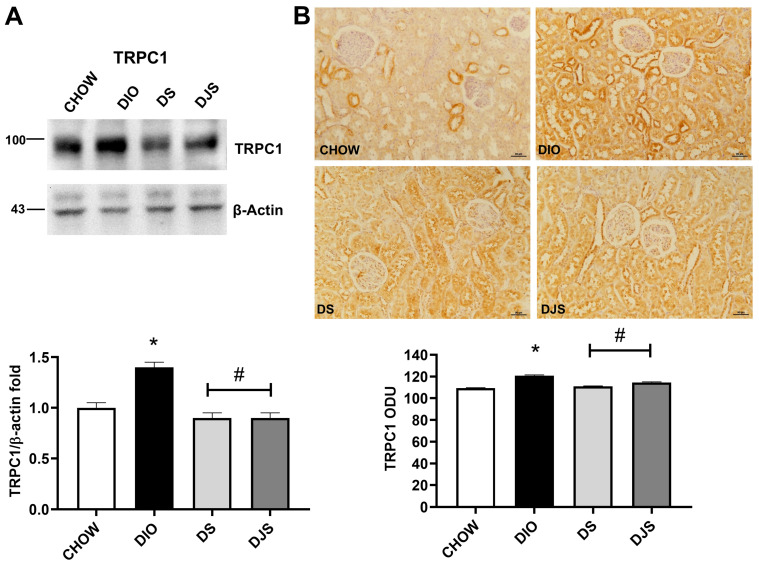
Transient receptor potential cation channel expression. (**A**) Kidney lysates were immunoblotted with anti-transient receptor potential canonical 1 (TRPC1). The graph shows the densitometric ratios of bands and β-actin expression, used to normalize the data. (**B**) Immunohistochemical representative pictures of kidney sections processed for TRPC1. Graphs indicate the mean intensities of the area immunoreaction of TRPC1. Scale bar 50 µm. CHOW, rats fed with standard diet; DIO, rats fed with high-fat diet; DS, DIO rats supplemented with tart cherry seeds; DJS, DS rats supplemented with tart cherry juice. Data are mean ± SEM. *: *p* < 0.05 vs. CHOW rats; #: *p* < 0.05 vs. DIO rats.

**Figure 7:**
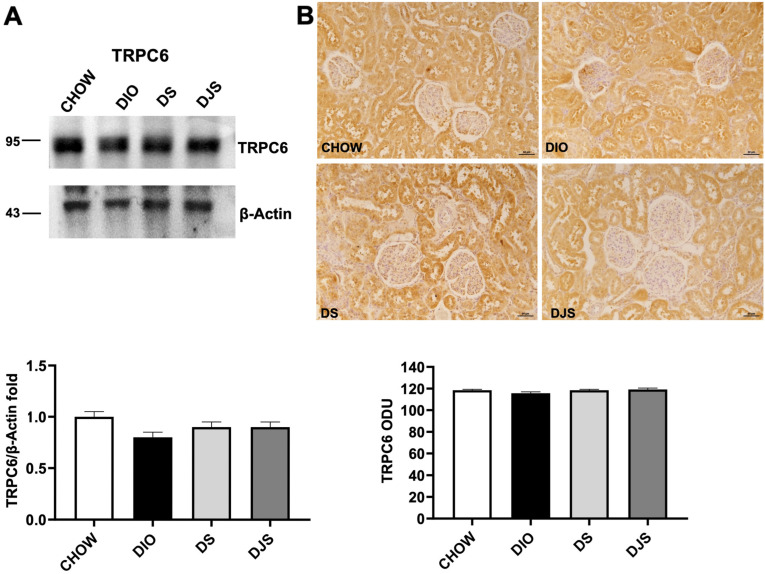
Transient receptor potential cation channel expression. (**A**) Kidney lysates were immunoblotted with anti-transient receptor potential canonical 6 (TRPC6). The graph shows the densitometric ratios of bands and β-actin expression, used to normalize the data. (**B**) Immunohistochemical representative pictures of kidney sections processed for TRPC6. Graphs indicate the mean intensities of the area immunoreaction of TRPC6. Scale bar 50 µm. CHOW, rats fed with standard diet; DIO, rats fed with high-fat diet; DS, DIO rats supplemented with tart cherry seeds; DJS, DS rats supplemented with tart cherry juice. Data are mean ± SEM.

**Figure 8:**
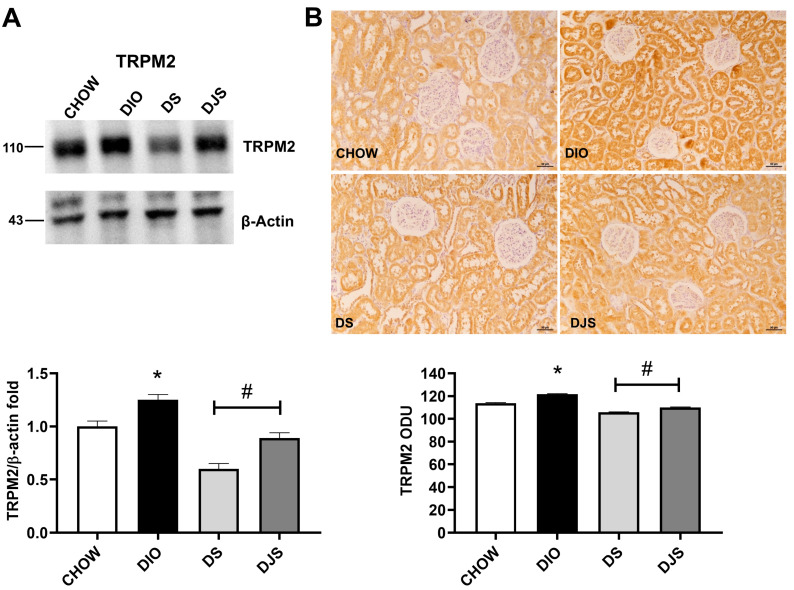
Transient receptor potential cation channel expression. (**A**) Kidney lysates were immunoblotted with anti-transient receptor potential melastatin 2 (TRPM2). Graph shows the densitometric ratios of bands and β-actin expression, used to normalize the data. (**B**) Immunohistochemical representative pictures of kidney sections processed for TRPM2. The graphs indicate the mean intensities of the area immunoreaction of TRPM2. Scale bar 50 µm. CHOW, rats fed with standard diet; DIO, rats fed with high-fat diet; DS, DIO rats supplemented with tart cherry seeds; DJS, DS rats supplemented with tart cherry juice. Data are mean ± SEM. *: *p* < 0.05 vs. CHOW rats; #: *p* < 0.05 vs. DIO rats.

For the WB, the incubation of membranes with anti-TRPC1, anti-TRPC6, and anti-TRPM2 antibodies revealed the following molecular weights: 100 kDa, 95 kDa, and 110 kDa, respectively (Figure 6A, Figure 7A and Figure 8A). An increased expression of TRPC1 was visible in the DIO rats compared to the CHOW control; a decrease in the DS and DJS groups was reported compared to rats fed a HFD without supplementation (Figure 6A). Following the WB, the IHC showed that TRPC1 was expressed at the level of the distal convoluted tubule with a significant increase in expression in the obese rats both in the tubular portion and glomerulus; a decrease was also observed in the DS and DJS groups (Figure 6B).

On the contrary, no remarkable difference was found in TRPC6 expression among the different groups, neither in WB (Figure 7A) nor in IHC (Figure 7B).

Significant differences were found in TRPM2 expression. The intensity of the lines, normalized for the corresponding reference protein expression, showed an increase in DIO rats compared to CHOW rats and a decrease with tart cherry supplementation, especially in the DS group (Figure 8A). Immunoreaction demonstrated that TRPM2 was expressed on the distal and convoluted tubule, but not in the glomerulus. A clear increase in the immunoreaction was detected in DIO rats compared to CHOW, while the density of immunoreaction decreased in DS and DJS compared to DIO rats (Figure 8B).

## 4. Discussion

Obesity is a complex, multifactorial disease that has reached pandemic levels across the world, and represents a major medical challenge due to its strong association with the development of chronic diseases, such as structural and functional kidney alterations promoting CKD. Although the prevalence, clinical manifestations, and pathophysiology of histopathological changes collectively referred to as obesity-related glomerulopathy have been reviewed [1,2,3,4], the molecular mechanisms have yet to be fully elucidated. Epidemiology evidence supports an augmented incidence of acute renal pathology in humans consuming HFD. Indeed, HFD intake has been found to induce renal lipotoxicity and metabolic dyshomeostasis [30], as well as glomerulopathy and proximal convoluted tubule injury, compromising the vital functions of the kidney [31]. Different evidence suggests that obesity contributes to CKD through mechanisms that involve chronic inflammation, hemodynamic alterations, insulin resistance, and lipid accumulation [4]. It has been proposed that the pathophysiology of obesity-related kidneys may involve a maladaptive response to caloric excess or a combination of maladaptive processes that causes damage via hemodynamic, hormonal, and lipotoxic pathways [32].

Comparable histological features, with progressive collapse of Bowman’s space, have been observed early in patients affected by diabetic nephropathy, leading to glomerulosclerosis in later stages of the disease [33], and in animal models subjected to HFDs [34,35,36,37,38]. In our study, the DIO model led to hypertension in rats, accompanied by adverse cortical renal damage with glomerulosclerosis and tubular atrophy, indicating a condition of renal disease. Hypertension is the main baseline disease contributing to CKD, especially when accompanied by proteinuria [39]. In the early stages of obesity, glomerular hyperfiltration acts as a compensatory mechanism to preserve sodium balance in response to exacerbated tubular reabsorption [40]. However, in the long term, these changes, along with increased blood pressure, created a hemodynamic burden on the kidneys that causes glomerular injury, such as renal hypertrophy. Prolonged obesity results in a gradual and progressive decline in nephron function, which further exacerbates hypertension [41]. In DIO rats, we demonstrated glomerulosclerosis in the absence of glomerular hypertrophy, which is consistent with previous studies in which the development of such obesity-related glomerulosclerosis can occur independently of glomerular hypertrophy and hyperfiltration [42].

Following the existing literature, we speculated that obesity status contributes to kidney injury through oxidative stress and oxidative-stress-associated inflammation—both recognized as key events by which chronic hyperglycemia causes renal cellular damage [43]. Current research has proven that dietary supplementations can improve intracellular protection against renal diseases [30]. In this context, a narrative review highlighted the nephroprotective role of anthocyanins [44]. For instance, dietary supplementation of glutathione was reported to provide protective effects against diabetic-kidney-associated pathologies [45]. As demonstrated in other animal models of obesity, the rise in the glutathione pool was involved in the renal-protective effects of anthocyanins [46]. In patients undergoing hemodialysis, the consumption of red fruit juice rich in polyphenol and anthocyanin contents increased the level of glutathione while also reducing DNA oxidation damage and protein and lipid peroxidation [47]. The nephroprotective properties of anthocyanins can be explained by their strong antioxidant and anti-inflammatory effects, as demonstrated in the rat models where bilberry extract was used to counteract the nephrotoxic effects of carbon tetrachloride [48]. Furthermore, the combination of hyperuricemia and hyperglycemia was a possible therapeutic target for renoprotection conferred by anthocyanins. This concept was reinforced by results from in vitro, in vivo, and clinical endocrinology studies proposing that mechanisms involving gut dysbiosis, renal nicotinamide adenine dinucleotide phosphate oxidase 4, nuclear factor erythroid 2-related factor 2, endoplasmic reticulum stress, and the NOD-like receptor protein 3 inflammasome may represent key molecular targets through which anthocyanins mitigate uric-acid-induced kidney injury [49]. Natural antioxidant compounds, such as curcumin, have demonstrated partial renoprotection and a favorable renal outcome after administration in diabetic rats with acute kidney injury [50]. Our data assessed a decrease in protein oxidation in the kidneys of obese rats fed with tart cherries, which was associated with attenuated disease severity and ameliorated renal histological features in DIO rats. The abnormalities of glucose metabolism in DIO rats were improved by tart cherry seeds and juice, as evidenced by decreased glucose levels and lowered blood pressure. These results indicate that the reduction in glucose levels may contribute to the renoprotective effects of tart cherries in obese rats, as previously demonstrated in ob/ob rats supplemented with anthocyanins [46]. However, the specific mechanism by which tart cherries exhibit antioxidative activity in the kidney remain unclear in the present study. Future research will be needed to elucidate the direct molecular target of tart cherry supplementation and to identify its potential effects on other obesity-related kidney complications.

Besides glucose metabolic dysfunction, lipid alteration was also implicated in the progression of kidney disease [51,52]. Previously, we found that tart cherry supplementation significantly decreased serum triglyceride content. These findings indicated that tart cherries exhibit a potent hypolipidemic effect, which may be involved in renal protection, even though this effect appears to be independent of any reduction in weight gain or white adipose tissue deposition. The adipose tissue in obesity secreted pro-inflammatory cytokines, such as IL-1β and IL-6, while simultaneously impairing the secretion of anti-inflammatory adipokines like adiponectin. This promoted the inflammatory response and insulin resistance, and caused the corresponding kidney injury [36,53]. Moreover, obesity-related nephropathy was further associated with regenerative cell proliferation, monocyte infiltration, and higher renal expression of monocyte chemotactic protein, leptin receptor, in addition to IL-6 [54]. IL-6 promoted growth and proliferation of mesangial cells, glomerular basement membrane thickening, and glomerulosclerosis, all of which contribute to the development of diabetic kidney disease. Meanwhile, IL-1α/β were involved in the impairment of inter-glomerular hemodynamic synthesis of prostaglandin by mesangial cells [7]. In line with the findings of Madduma Hewage et al. [55], we observed an increase in pro-inflammatory cytokines in the kidneys of obese animals. Here, we demonstrated that the glomerulosclerosis in the kidneys of DIO rats was not primarily driven by apoptosis, but rather by inflammation and oxidative stress due to HFD consumption.

This conclusion is consistent with findings in other organs, such as the liver [23], heart [27], and brown adipose tissue [28], in which the presence of histopathological alterations was not related to caspase 3 activation. In addition, we found that supplementation with tart cherries reduced the renal levels of IL-1β and IL-6, indicating an attenuation of inflammation. The anti-inflammatory activities of tart cherries, particularly those of the seeds, may contribute to their protective effects against CKD. In accordance with several studies conducted in cell lines and in overweight and obese subjects in a randomized, crossover pilot study testing both tart cherry extract and juice [56,57,58], we confirmed their antioxidant and anti-inflammatory effects in mitigating inflammation and oxidative stress in other organs. Similarly, anthocyanins extracted from purple corn inhibited diabetes-associated glomerular monocyte activation and macrophage infiltration [59], as well as dampened high-glucose-induced mesangial fibrosis and inflammation, showing a possible renoprotective role in diabetic nephropathy [60]. Moreover, dietary supplementation of lingonberries, which are rich in anthocyanins, not only attenuated the HFD-induced renal inflammation but also reduced kidney injury in obese mice [55]. This supplementation enhanced plasma lipid and glucose levels, lowered the amount of cytokine in the plasma, without modified body weight gain raised from HFD. Extract of lingonberry as well as its active constituent cyanidin-3-glucoside efficiently repressed palmitic acid-induced nuclear factor kappaB (NF-kB) stimulation and inflammatory cytokine level in proximal tubular cells. These outcomes advised that lingonberry supplement diminished the inflammatory answer and stopped chronic kidney damage [55].

The complex role of the TRP family in diabetes and obesity has been recently reviewed by Moraes et al. [61]. Dysfunctions in some members of the TRP family have been implicated in obesity, hypertriglyceridemia, diabetes, and hypertension. However, the literature presents heterogeneous findings, which may be attributed to several factors, including differences in the metabolic profiles of diabetic animals, dissimilar phases of diabetes, and the kind of arteries and veins examined. However, the in vivo significance of these conclusions has not been revealed [61]. To date, the function of TRPCs in obesity remains largely unexplored. In line with this variability, we previously found a different modulation of TRPC1, TRPC6, and TRPM2 in the hippocampus and frontal cortex of DIO rats [25]. We also observed that TRPC1 and TRPM2 were upregulated in the kidneys of obese animals; however, no significant differences were found for TRPC6. We confirmed TRPC6 expression in both the glomeruli and in the proximal and distal tubular portion by immunohistochemical analysis. In contrast, TPRPC1 and TRPM2 were particularly expressed at the levels of the tubules [14]. In fact, TRP channels are known to influence renal pathology by promoting unfavorable calcium influx, increasing oxidative stress and inflammatory processes. Their roles are especially marked in conditions like ischemia and nephrotoxicity, which induce fibrosis and nephron loss in CKD [62].

TRPC1 inhibits the positive effect of exercise on type 2 diabetes risk under a HDF-induced obesity environment and plays an important role in the regulation of adiposity via autophagy and apoptosis [11]. An association among TRPC1 disfunction and diabetic nephropathy has also been proposed in animal models [12]. One study suggested that TRPC1 was elevated in a porcine model of metabolic syndrome produced by feeding 6–9-month-old pigs a HFD [63]. On the contrary, TRPC1 expression was found to be reduced in diabetes [64], but any causal relationship was unknown. Recently, it was concluded that the *TRPC1* gene regulates body metabolism and that, except for hypertension, phenotypes of mice after deletion of the *TRPC1* gene resembled mice with metabolic syndrome [65]. In the pathophysiology of non-genetic forms of kidney disease, such as diabetic nephropathy, focal segmental glomerulosclerosis, renal fibrosis and immune-mediated kidney diseases, TRPC6 has been involved, and thus, it is considered as a significant marker for the advance of therapeutic mediators to treat various kidney pathologies [13,66]. TRPC6 inactivation in rats resulted in the protection of glomeruli in the animal model of severe glomerulonephritis. However, its role in renal fibrosis appeared to be model-dependent and possibly species-dependent [67]. An up-regulation of TRPC6 channels has been reported in renal ischemia–reperfusion injury, where they seem to be involved in the podocyte response to renal damage [68] and in diabetic nephropathy tissues and HK-2 cells cultured in high glucose concentrations [69]. However, confounding genomic and non-genomic effects of other TRPC channels should be taken into consideration to fully comprehend the therapeutically renoprotective potential of targeting TRPC6 under chronic kidney-damaging conditions [70]. In addition, it was reported that TRPC6 did not play a role in the acute phase of acute kidney injury [71]. An increasing number of works have also suggested that TRPC6 is responsible for tightly regulating the immune cell functions; however, it remains unclear whether the role of TRPC6 in the immune system and the pathogenesis of renal inflammation are intertwined [72]. While several TRP channels expressed in the kidney were involved in disease states, such as those implicated in polycystic kidney disease and focal segmental glomerulosclerosis [15,16], the role of TRPM2 in kidney physiology or pathophysiology is still unknown. However, it was demonstrated that TRPM2 mediated ischemic kidney injury and oxidant stress [14]. Interestingly, *TRPM2*-deficient mice were protected from developing diet-induced obesity and insulin resistance when fed a HFD [73]. These mice presented an intensification in insulin sensitivity, while at the same time improved skeletal muscle and cardiac glucose metabolism were detected which subsequent in overall augmented energy effort. The current indication focused the attention on a key function of TRPM2 in the pathophysiology of diabetes and further metabolic disorders such as obesity [74,75]. TRPM2 was an important moderator in ischemic acute kidney injury, and the deletion of TRPM2 and pharmacological blockade of TRPM2 could produce protection in the kidney [75]. The fundamental mechanisms implicated mostly the activation of the nicotinamide adenine dinucleotide phosphate oxidase complex component RAC1 and the subsequent rise of oxidative stress, as well as initiation of apoptotic paths in the mitochondria. Additionally, RAC1 inhibition before ischemia helped to preserve kidney function [75]. Of most interest in the present study is the interaction between tart cherries, which are rich in anthocyanins, and TRP channels. This relationship, to our knowledge, has not been previously investigated, and constitutes the key innovative contribution of this work. Notably, both seed and juice supplementation attenuated the alterations reported in the DIO model. Thus, taken together with previous evidence, our findings suggest that TRP channel modulation by anthocyanins could represent another important and novel mechanism underlying the renal protection related to the anti-inflammatory and antioxidant effects of tart cherries.

In fact, the antioxidant properties of tart cherry could modulate the calcium ion influx related to the increase in TRPM2; these could also counteract cellular damage in the kidney tissues, as well as apoptosis and necrosis which are exacerbated by this influx [62,76]. The modulation of TRPM2 by tart cherry could be explained by the observed reduction in renal fibrosis and inflammation in obese rats. TRPM2 activation influences the deposition of fibrotic tissue in the renal parenchyma, regulating the activity of transforming growth factor β1. Further complicating its role, TRPM2 activation also contributes to the inflammation enhancing NF-κB signaling, which in turn leads to the up-regulation of inflammatory cytokines, such as tumor necrosis factor-α, IL-1β, and IL-6 [77,78].

## 5. Conclusions

This study demonstrated that tart cherry supplementation attenuated kidney glomerulosclerosis, fibrosis, protein oxidation, inflammation, and TRP channel alterations in rats fed a HFD. The present study indicated the contribution of inflammatory processes and oxidative stress in the development of obesity-related renal damage. Further research is required to characterize the functional correlations of kidney damage, particularly regarding glomerular filtration and tubular reabsorption efficacy. The observed protective effects of anthocyanin-rich tart cherry supplementation provide promising findings for more in-depth investigations into its potential dietary integration to prevent obesity-induced renal damage. Future investigations should also focus on the pharmacokinetics and pharmacodynamics of its various bioactive components.

## Figures and Tables

**Table 1:** Morphological parameters of kidneys.

	CHOW	DIO	DS	DJS
Kidney weight (g)	2.2 ± 0.1	1.9 ± 0.1	2.0 ± 0.1	1.9 ± 0.1
Capsular volume 10^3^ µm^3^	22.1 ± 0.4	21.6 ± 0.8	21.1 ± 1.4	20.7 ± 0.5
Glomerular tuft volume 10^3^ µm^3^	16.5 ± 0.3	15.7 ± 0.5	14.9 ± 0.9	15.5 ± 0.2
Glomerular injury score (GIS)	1.0	1.4 ± 0.1 *	1.3 ± 0.2	0.9 ± 0.1 #

Data are the mean ± SEM; * *p* < 0.05, vs. CHOW rats # *p* < 0.05 vs. DIO rats.

## Data Availability

The data presented in this study are available upon request from the corresponding author.

## Supplementary Materials

The following supporting information can be downloaded at: https://www.mdpi.com/article/10.3390/antiox14091102/s1, Supplementary File S1: Additional materials and methods containing more detailed protocols.

## Abbreviations

The following abbreviations are used in this manuscript:

CKD	Chronic kidney disease
DIO	Diet-Induced Obesity
GIS	Glomerular injury score
HFD	High-Fat-Diet
IHC	Immunohistochemistry
IL-1 β	Interleukin-1 β
IL-6	Interleukin-6
PAS	Periodic Acid–Schiff
SEM	Standard error of mean
TRP	Transient receptor potential
TRPC1	Transient receptor potential canonical 1
TRPC6	Transient receptor potential canonical 6
TRPM2	Transient receptor potential melastatin 2
WB	Western Blot
