# Supplementary material for: High-Fat-Diet-Induced Kidney Injury in Rats: The Role of Tart Cherry Supplementation
Source: Antioxidants (Basel). 2025 Sep 10;14(9):1102. doi: 10.3390/antiox14091102 (PMC12466484; doi:10.3390/antiox14091102)
Supplement: Supplementary file 1 [file antioxidants-14-01102-s001.zip › antioxidants-3780186-supplementary.pdf]

Supplementary 1.
Additional materials and methods containing more detailed protocols.

Animals: diet, general and blood parameters

Male Wistar rats (Charles River; total n = 44; 225–250 g) were used. Sample size was calculated using G*power analysis, based on previous experiments conducted in our laboratory and others on the same obesity animal model. Rats were randomly divided into two groups with no significant differences in initial mean body weight. The first group (n = 8) was fed with standard laboratory chow ad libitum (4RF18, Mucedola, Settimo Milanese, Italy; 2.6 kcal/g), called CHOW rats. The second group (n = 36), referred to as DIO rats was fed *ad libitum* with high-fat-diet (HFD; 45% fat, 35% carbohydrate, 20% protein; D12451, Research Diets, Inc., New Brunswick, NJ; 4.73 kcal/g). This DIO group was further subdivided into 3 groups (n = 12 each):

- DIO control rats (n = 12): received only HFD.
- DS rats: received HFD plus supplementation of *Prunus Cerasus* L. seed powder 0.1 mg/g/die.
- DJS rats: received HFD plus supplementation with the same dose of seed powder (0.1 mg/g/die) and juice containing 1 mg of anthocyanins.

The juice was administered daily; once the rats had completely consumed it, a bottle of water was placed in the cage. For both DS and DJS rats, the seed powder was mixed into 1 g of lard (selected for its high palatability) and placed in the cage in a special bowl. DIO controls animals received the same amount of lard bowl without tart cherry seed powder.

6 of the 36 rats fed with HFD were excluded from the study because they did not significantly increase body weight compared to CHOW rats.

Tart cherry supplementation did not modify the weight gain. Here, the body weight values (g): CHOW 557.0 ± 10.7; DIO 682.8 ± 17**; DS 683.1 ± 29.7**; DJS 689 ± 20.8** (**$p < 0.01$ *vs.* CHOW rats).

For the blood parameters the different groups showed the following values:

-Systolic blood pressure (mmHg): CHOW 110.9 ± 6.1; DIO 140.3 ± 8.1*; DS 111.4 ± 5.5#; DJS 107.6 ± 6.01#.

-Glycemia (mg/dL): CHOW 91.6 ± 5.1; DIO 126.8 ± 6.1*; DS 105.7 ± 3.6#; DJS 111.3 ± 2.4*#.

-Insulin (μg/L): CHOW 0.73 ± 0.05; DIO 1.06 ± 0.05*; DS 1.01 ± 0.06*; DJS 1.03 ± 0.06*.

-Cholesterol (mg/dL); CHOW 76.1 ± 3.3; DIO 75.6 ± 4.1; DS 69.8 ± 5.6; DJS 77.6 ± 4.4.

-Triglycerides (mg/dL): CHOW 76.6 ± 10.4; DIO 84.3 ± 13.9; DS 42.9 ± 3.6*#; DJS 49.8 ± 1.9*#

-Leptin concentration (pg/mL): CHOW 3449.7 ± 165.6; DIO 9159.2 ± 110.1*; DS 8362.6 ± 184.5*#; DJS 7572.0 ± 65.9*#

(*p<0,05 *vs* CHOW rats; #p<0,05 *vs* DIO rats)

These information's were already published elsewhere [21-24,27,28].

Tart Cherry seeds and Juice preparation: total phenolic compound and anthocyanin determination

Tart cherries were provided by Azienda agricola Sigi Sas (Macerata, Italy). The preparation of seed powder and juice from tart cherries has also been previously described in our studies [21,23,24,27]. In detail, seeds were removed from the cherries, and the pulps were homogenized. The resulting homogenate was then centrifuged, and the supernatant (pulp extract) was removed and stored at 4 °C. The remaining precipitate (cherry residue) was further extracted with ethanol. This ethanol solution was centrifuged, and the supernatant (ethanol extract) was collected and evaporated with a Rotavapor to remove the ethanol. The concentrated juice was added to the initial pulp extract and standardized; in this way, the rats received a daily dose of 1 mg of anthocyanins every day for 17 weeks [22]. The amount of total phenolic compounds in tart cherry samples was determined spectrophotometrically according to the Folin–Ciocalteu method, using gallic acid as a calibration standard. After 10 min at 37 °C, 50 μL of a saturated solution of sodium carbonate was added. The absorbance of each solution was revealed at 765 nm. The total amount of phenolics was calculated as mg Gallic Acid Equivalent (GAE)/g sample. The total monomeric anthocyanin content was measured by the pH differential method [22,28] and expressed as cyanidin-3-glucoside equivalents per liter, using a molar absorptivity of 26,900 L/mol and a molecular weight of 449.2 g/mol. The total amount of phenolic content was found to be 23 ± 1 GAE/g sample, and the total anthocyanins were 0.546 mg/mL.

The preparation of seed powder and juice from tart cherries has already been described in our previous studies [21-24,27]. As detailed elsewhere [22], the fatty acid composition of ultrasound-assisted extraction (UAE) defatted seeds was distributed in the following percentages:

- Linoleic (18:2) 44.08%;
- oleic (18:1) 43.30%;
- Palmitic (16:0) 6.70%;
- Stearic (18:0) 4.14%;
- Eicosanoic (20:0) 1.05%;
- Palmitoleic (16:1) 0.31%;
- Eicosenoic (20:1) 0.42%.
